# Improving pairwise comparison of protein sequences with domain co-occurrence

**DOI:** 10.1371/journal.pcbi.1005889

**Published:** 2018-01-02

**Authors:** Christophe Menichelli, Olivier Gascuel, Laurent Bréhélin

**Affiliations:** 1 IBC, LIRMM, Univ. Montpellier, CNRS, Montpellier, France; 2 Unité de Bioinformatique Evolutive, C3BI - USR 3756, Institut Pasteur et CNRS, Paris, France; Dassault Systemes BIOVIA, UNITED STATES

## Abstract

Comparing and aligning protein sequences is an essential task in bioinformatics. More specifically, local alignment tools like BLAST are widely used for identifying conserved protein sub-sequences, which likely correspond to protein domains or functional motifs. However, to limit the number of false positives, these tools are used with stringent sequence-similarity thresholds and hence can miss several hits, especially for species that are phylogenetically distant from reference organisms. A solution to this problem is then to integrate additional contextual information to the procedure. Here, we propose to use domain co-occurrence to increase the sensitivity of pairwise sequence comparisons. Domain co-occurrence is a strong feature of proteins, since most protein domains tend to appear with a limited number of other domains on the same protein. We propose a method to take this information into account in a typical BLAST analysis and to construct new domain families on the basis of these results. We used *Plasmodium falciparum* as a case study to evaluate our method. The experimental findings showed an increase of 14% of the number of significant BLAST hits and an increase of 25% of the proteome area that can be covered with a domain. Our method identified 2240 new domains for which, in most cases, no model of the Pfam database could be linked. Moreover, our study of the quality of the new domains in terms of alignment and physicochemical properties show that they are close to that of standard Pfam domains. Source code of the proposed approach and supplementary data are available at: https://gite.lirmm.fr/menichelli/pairwise-comparison-with-cooccurrence

## Author summary

Deciphering the functions of the different proteins of an organism constitutes a first step toward the understanding of its biology. Because they provide strong clues regarding protein functions, domains occupy a key position among the relevant annotations that can be assigned to a protein. Protein domains are sequential motifs that are conserved along evolution and are found in different proteins and in different combinations. One common approach for identifying the domains of a protein is to run sequence-sequence comparisons with local alignment tools as BLAST. However these approaches sometimes miss several hits, especially for species that are phylogenetically distant from reference organisms. We propose here an approach to increase the sensitivity of pairwise sequence comparisons. This approach makes use of the fact that protein domains tend to appear with a limited number of other domains on the same protein (the domain co-occurrence property). On *P. falciparum*, our approach allows identifying 2240 new domains for which, in most cases, no domain of the Pfam database could be linked.

This is a *PLOS Computational Biology* Methods paper.

## Introduction

Proteins are macromolecules essential for the structuring and functioning of living cells. Proteins generally have different functional regions which are conserved along evolution [1] and are commonly termed as “functional motifs” or “domains”. Domains/motifs are found in different proteins and combinations [2] and, as such, are functional protein subunits above the raw amino-acid level. Domain identification is thus an essential task in bioinformatics.

Different strategies can be used to identify these regions of a target protein. Profile analysis, also known as sequence-profile comparison, is a powerful method. This *non ab initio* method requires a database of protein domains, Pfam being one of the most widely used [3]. In Pfam, each family of domains is defined from a manually selected and aligned set of protein sequences, which is used to learn a profile hidden Markov model (HMM) of the domain [4]. To identify new protein domains, each HMM of the database is used to compute a score that measures the similarity between the sequence and the domain. If the score is above a predefined threshold, the presence of the domain in the protein can be asserted. However this method may miss several domains when applied to an organism that is phylogenetically distant from the species used to train the HMM. This may happen for two reasons. First, if the protein sequence has encountered many evolution events, the HMM of the database may poorly fit the sequence specificity of the distant organism. Different approaches can be used in this case. One approach is to use co-occurrence properties of protein domains (see below) [5–10]. Another approach is to fit the HMMs to the sequence specificities of the target species. Terrapon et al. [11] propose a few solutions to this problem. Finally, another approach is to analyze the pattern of hydrophobic amino-acids in the sequence. Because hydrophobic clusters are more conserved than sequence itself, they can be used as signatures for comparing sequences. Hydrophobic clusters analysis have shown to be a valuable tool for identifying domains in remote sequences [12, 13]. Another possibility that can explain missing domains is that those domains belong to families that are simply absent from the domain database. Databases like Pfam were built with eukaryotic sequences that originate mostly from plants, fungi and animals, and very few from the other groups. Hence, the proportion of proteins covered by a Pfam domain in plants, fungi and animals is around twice that found in the other super-groups on the eukaryote tree (Chromalveolates and Excavates) [7]. For example, in *Plasmodium falciparum*, which is the organism responsible for the deadliest form of malaria, only 22% of its protein residues are covered by a Pfam domain while this percentage is as high as 44% for both yeast (*Saccharomyces cerevisiae*) and humans.

When some domains of the target proteins are absent from the domain database, an alternative approach for their identification is to run an *ab initio* approach based on sequence-sequence comparison using pairwise comparison tools like FASTA [14] or BLAST [15]. These tools look for local similarities between a query protein and a sequence database like Uniprot [16]. Because domains are sub-sequences conserved throughout evolution, local similarities between proteins usually correspond to these regions. Tools like BLAST use specific scoring functions for assessing similarities. Moreover, they provide estimates of p-values (and e-values) under specific hypothesis of score distribution. Sequence-sequence approaches do not include information from other homologous sequences, so they are more prone to false negatives/positives than sequence-profile approaches. Therefore, they are usually used with stringent score thresholds to avoid false positives, and hence they may also miss several homologies. Different versions of BLAST were developed to improve the sensitivity. For example, PSI-BLAST [17], which constructs a position-specific score matrix (PSSM) to perform incremental searches, PHI-BLAST [18], which uses a motif to initiate hits, or DELTA-BLAST [19], which searches a database of pre-constructed PSSMs before searching a protein-sequence database to yield better homology detection.

**Fig 1:**
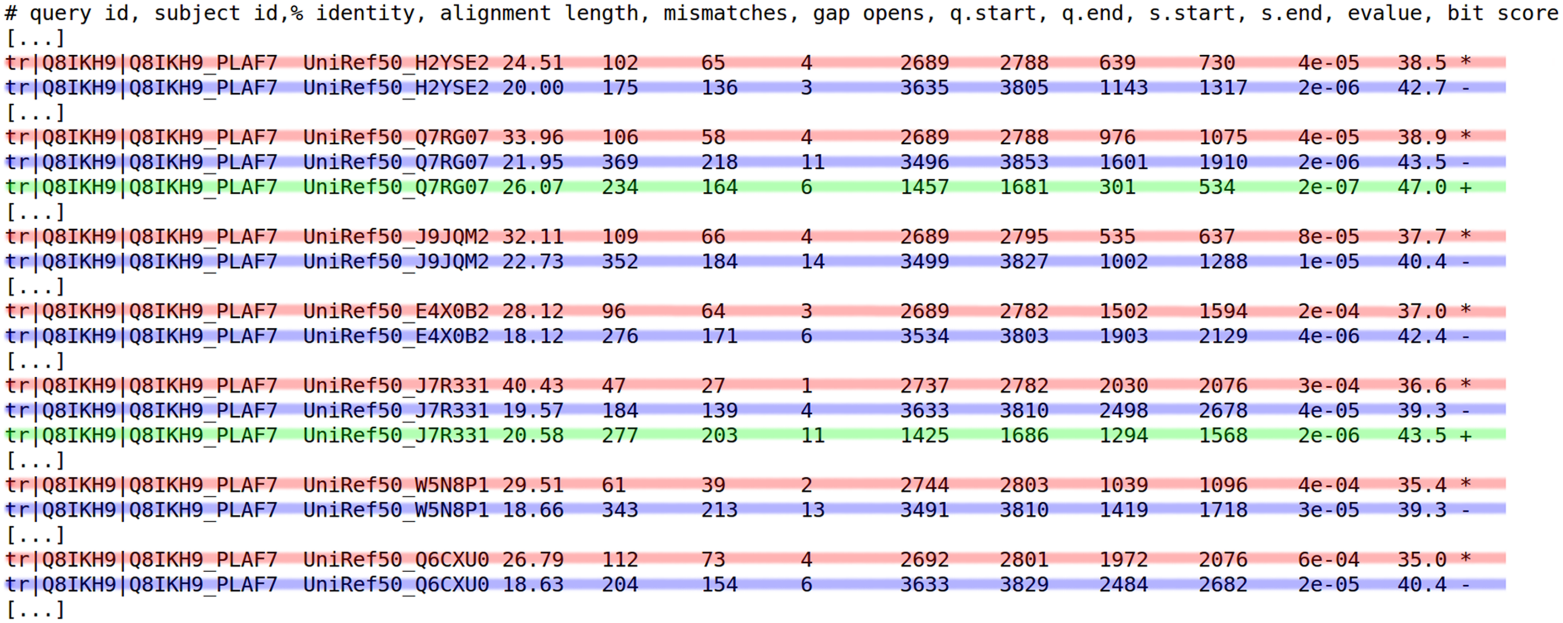
Extract of BLAST results on query sequence Q8IKH9_PLAF7 on UniRef50 (fields: query id, subject id, % identity, alignment length, mismatches, gap opens, q. start, q. end, s. start, s. end, e-value, bit score). Note that some hits are hidden for clarity. Depending on the target protein, the BLAST result reveals the co-occurrence of two/three independent sub-sequences (each sub-sequence is highlighted with a different color).

Surprisingly, so far domain co-occurrence has not been used to improve the sensitivity of sequence-sequence approaches in proteins. Domain co-occurrence is a strong feature of proteins, based on the fact that many protein domains tend to appear with a limited number of other domains on the same protein [2]. A well known example are domains PAZ and PIWI, which are frequently found together: when assessing proteins with the PAZ domain, the PIWI domain is frequently found. Functional studies have shown that domains that co-occur in proteins are more likely to display similar functions than domains that appear in separate proteins [20]. Co-occurrence information has already been used for improving the sensitivity of sequence-profile approaches [5, 8–10]. However, it could also be of great help for sequence-sequence homology detection. For example, Fig 1 reports homologies found between a *Plasmodium falciparum* protein and three proteins from Uniprot. Most of these hits have moderate e-values and, taken independently, cannot be considered with high confidence. However, every hit actually co-occurs with one or two other hits on the same protein, and these co-occurrences are present in all three proteins. Taken altogether, this information adds strong evidence on the identified homologies.

Here we propose a new method to take co-occurrence into account in a typical BLAST analysis and to construct new domain families based on these results. Our procedure is based on the analysis of co-occurring hit density along the query protein. We designed a procedure that uses this density to identify hit clusters that sign domain boundaries. We present our approach and propose a statistical test to assess the relevance of the identified clusters. Finally, we apply our approach to the entire *P. falciparum* proteome and show that, on this organism, it allows us to increase the number of significant hits by 14%. Moreover, our procedure for identifying new domain families enables us to increase proteome area that can be covered with a domain by 25%.

## Results

### Computational approach

**Fig 2:**
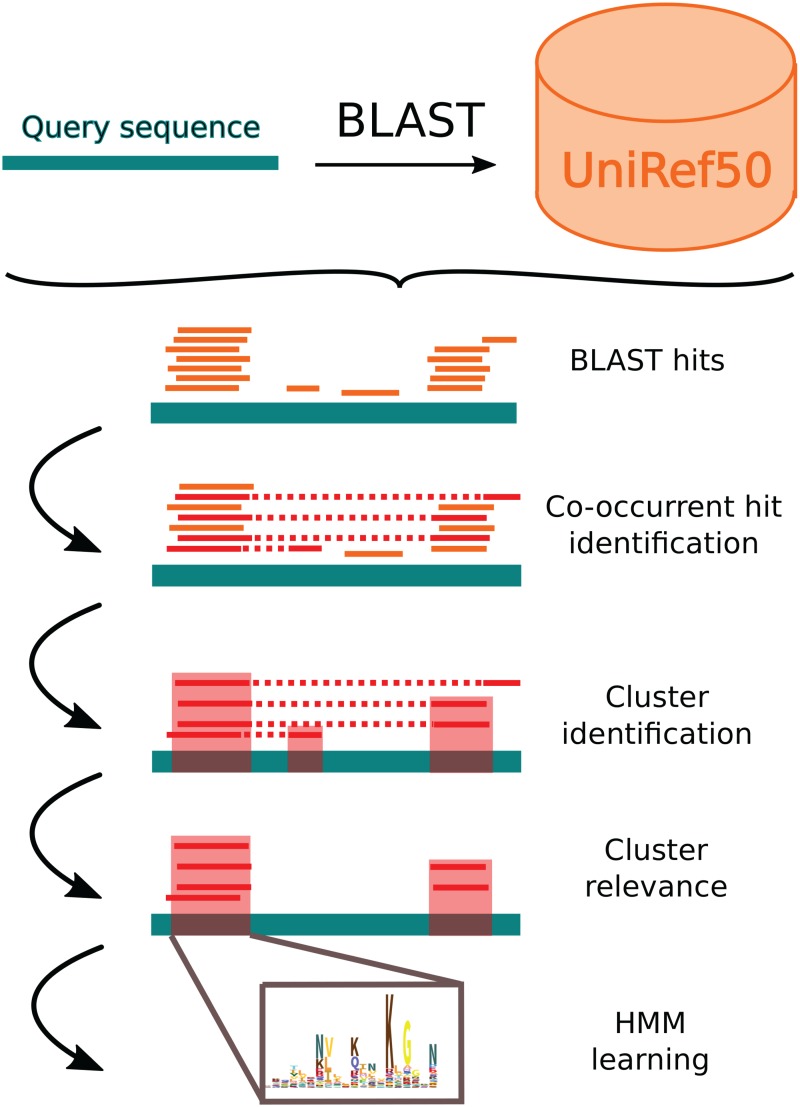
Flowchart of the main steps of the procedure.

Our aim is to improve the sensitivity of pairwise comparison tools such as BLAST using co-occurrence information. The core of our approach is a new scoring function that takes co-occurrence information into account for assessing BLAST hits. This new scoring function allows us to identify interesting hits that would not be considered solely on the basis of BLAST results because of too high e-values. A flowchart representing the main steps of the procedure is depicted on Fig 2.

#### Discovering domains from BLAST results

The initial step is to perform a BLAST search of the query protein against target sequences present in a protein sequence database. In the experiments below, we used the UniRef50 database and the BLASTP software package [15] with default parameters and a max e-value set at predefined cutoff (for example 10^−3^). This search gives us a list of all similarities found for the query sequence. Each pair of similar sub-sequences is called a hit. All hits smaller than 30 residues are removed, and in case of overlapping hits on a target protein only the hit with the lowest e-value is considered. Note that BLAST controls sequence complexity by automatically discarding low-complexity sub-sequences (default parameters).

**Fig 3:**
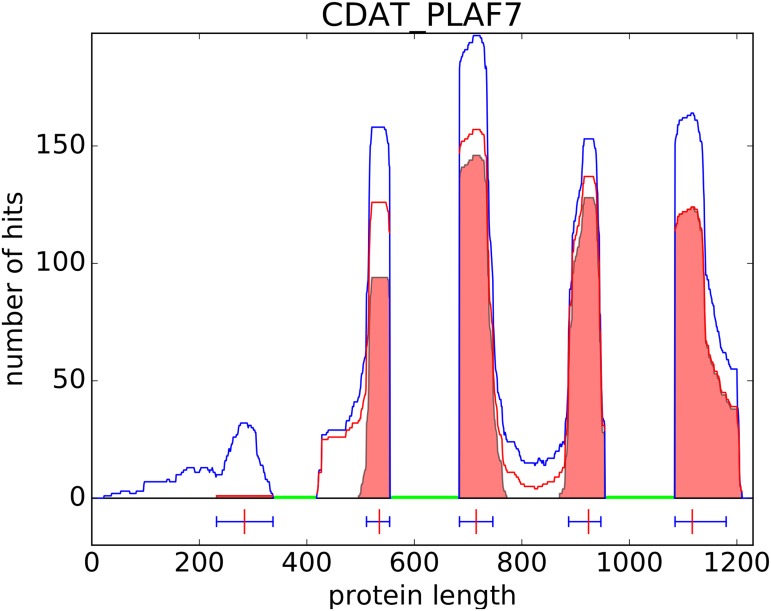
Density of BLAST hits per residue on the sequence CDAT_PLAF7. The blue line represents the density obtained using all hits. The red line represents the density obtained using only hits that have a co-occurring hit on the same protein. The filled regions in red show hit clusters identified by our method. Lines under the horizontal axis indicate positions of clusters (domain boundaries) identified by our procedure on this protein. In this example, regions already covered by a Pfam domain were masked (green regions) so that no hits were identified by BLAST.

Next we use the BLAST results to discover potential domains. Our hypothesis is that domains are the most conserved protein sub-sequences. Under this hypothesis, domains should correspond to regions with the highest number of hits in a classical BLAST search. Hence, we use the density of hits per residue to identify the potential domains of a given protein. The main drawback of this approach is that it can potentially produce many false positives. First, high peaks can also be due to low complexity regions that have not been properly masked by BLAST. Second, even if a peak corresponds to a domain, all hits that compose the peak are not necessarily true occurrences of this domain, and the proportion of contaminants can be high in certain cases. To avoid a maximum of false positives, our solution is to look at hits for which we have found a co-occurring hit. We define a co-occurring hit as another hit involving the same query and target proteins and that does not overlap with the former hit on any two proteins. Hence, rather than working on the hit density (blue curve in Fig 3), we work on the co-occurring hit density, i.e. the density of hits with a co-occurring hit (red curve in Fig 3). The goal is then to identify clusters of homologous hits that would represent protein domains. This is a difficult problem because all hits in a peak do not have the same length and are not perfectly aligned. Moreover, two adjacent domains on the query protein can also be adjacent in certain target proteins. In this case, the two domains would appear as a single long hit and may create ambiguity.

We developed an iterative heuristic for this problem. The method focuses on the co-occurring hit density, and starts by identifying the position associated with the highest peak (see second red peaks in Fig 3). All hits covering this specific residue are selected and used to define the boundaries of the domain. This is done by iteratively selecting an homogeneous subset of these hits, *i.e.* by incrementally removing hits whose begin and end positions are too far from the other selected hits (see Algorithm 1 in Methods for details). At the end of this process we identify a cluster of similar hits, *i.e.* similar in context because they share the co-occurrence property, and similar in sequence because they are located on the same region on the query protein. The cluster defines a protein domain family, with the different hits being different occurrences of the domain in different proteins. The region covered by this domain is then masked, and the whole procedure is resumed until no new domain can be identified on the protein.

#### Evaluating cluster relevance

Selecting only hits that have a co-occurring hit should allow us to avoid many false-positives. However, co-occurrence can also be detected simply by chance. To control this, for each identified cluster (domain family), we compare the number of hits with co-occurring hits (red curve) to the total number of hits that overlap this domain (blue curve), and we estimate the probability of observing as many co-occurring hits by chance. We use a binomial test for this purpose, and the p-value is computed as:

p-value=∑k=mn(nk)pk(1-p)n-k. (1)

*m* is the number of target proteins with a hit overlapping the domain and a co-occurring hit outside the domain; it is given by the red curve of Fig 3. *n* is the total number of target proteins with a hit overlapping the domain; it is given by the blue curve of Fig 3. *p* is the prior probability that a hit overlapping the domain has a co-occurring hit outside the domain, given the total number of hits outside the domain. This probability depends on the query protein and on the domain. For example, some proteins may have several low complexity regions with a lot of hits on UniRef50. In this case, it is easier for a hit to have co-occurring hits outside the domain. Prior probability *p* is estimated by the total number of proteins with a hit on the query protein outside the domain, divided by the total number of proteins in the database. It is conveniently computed by $\frac{N-n+m}{U}$, with *N* being the total number of proteins with a hit on the query protein and *U* the total number of proteins in the database (UniRef50). The number of proteins overlapping a domain are computed on the position with the highest density of hits. If several residues have the same density, the central position is selected. Domains with a p-value higher than a predefined threshold (for example 10^−3^) are discarded.

#### Estimating the number of false discoveries

The procedure described above is intended to be applied to all proteins of a given organism. For each discovered domain, the computed p-values allows us to check that the number of co-occurring hits cannot be found by mere chance. This ensures that the hits that compose a cluster likely occur from the same domain family. However, this does not ensure that the query protein is homologous to this particular family. Indeed, a protein may fortuitously possess two regions that resemble two domains that are frequently found together. In this case, we may observe many co-occurring hits, but the protein regions will not be homologous to the identified domain families. Although this should rarely happen, it is important to estimate the number of false positives detected by our procedure when it is applied to a whole proteome. We used two estimation procedures for this purpose. In the first procedure, each protein of the query organism was randomized by randomly shuffling the 4-mers that compose its sequence. In the second procedure, each protein sequence was simply reversed. Then, the whole procedure was resumed on these fake sequences (*i.e.* BLAST search, co-occurrence analysis, hits clustering, evaluation of cluster relevance), and the number of domains below the given p-value threshold was computed. By comparing the number of domains “identified” in fake data to the number of domains identified in real data, we can get an estimate of the proportion of false positives of our procedure:

$$\begin{array}{c} \text{FDR}=\frac{\text{number}\;\text{of}\;\text{discovered}\;\text{domains}\;\text{in}\;\text{fake}\;\text{data}}{\text{number}\;\text{of}\;\text{discovered}\;\text{domains}\;\text{in}\;\text{real}\;\text{data}}. \end{array}$$ (2)

In the following, we provide two FDR estimates: one FDR estimated on the randomized sequences (4-mers shuffling) and one FDR estimated on the reverse sequences.

#### Learning new models

Once new domain families have been identified with the above procedure, an additional and optional step is to learn an HMM for each cluster. Only clusters with at least 5 sequences are considered to learn a model. We first realigned the sequences of each cluster using MUSCLE software [21], with default parameters. Flanking positions with less than 75% residues were removed. Then, each multiple sequence alignment (MSA) was used to train a HMM representing the corresponding domain family using HMMER [22] (see Methods).

#### Integrating known domains into the procedure

In order to focus on regions that are not already covered by a known domain, an interesting improvement is to include known domain information in the procedure. This is done at two levels. First, prior to the BLAST search, regions already covered by a Pfam domain are masked by replacing the covered residues by the unknown residue (X) which is ignored by BLAST. Next, when searching for co-occurring hits, known Pfam domains are also considered as potential co-occurring hits. Namely, if a query protein A has a BLAST hit on a target protein B, and if both A and B have the same Pfam domain D (and that D does not overlap the considered hit on A and B), then the BLAST hit is considered as having a co-occurring hit. Integrating known domain information has two advantages. First, it enables us to speed up the BLAST search by masking part of the query sequences. Second, by integrating accurate homology information, it also minimizes the chance of detecting false positive co-occurrences and improves the quality of the results.

### Experiments on *P. falciparum* proteins

We applied our procedure to *Plasmodium falciparum* proteins. Each protein sequence (release February 21 2016 on Uniprot; 5365 proteins in total) was used to run a BLAST search against the UniRef50 database (October 2015) [23] restricted to eukaryotic sequences, which gathers 2784993 sequences from 2753 reference organisms with a maximum of 50% similarity between each pair of sequences. We used Pfam release 28 for these experiments.

**Fig 4:**
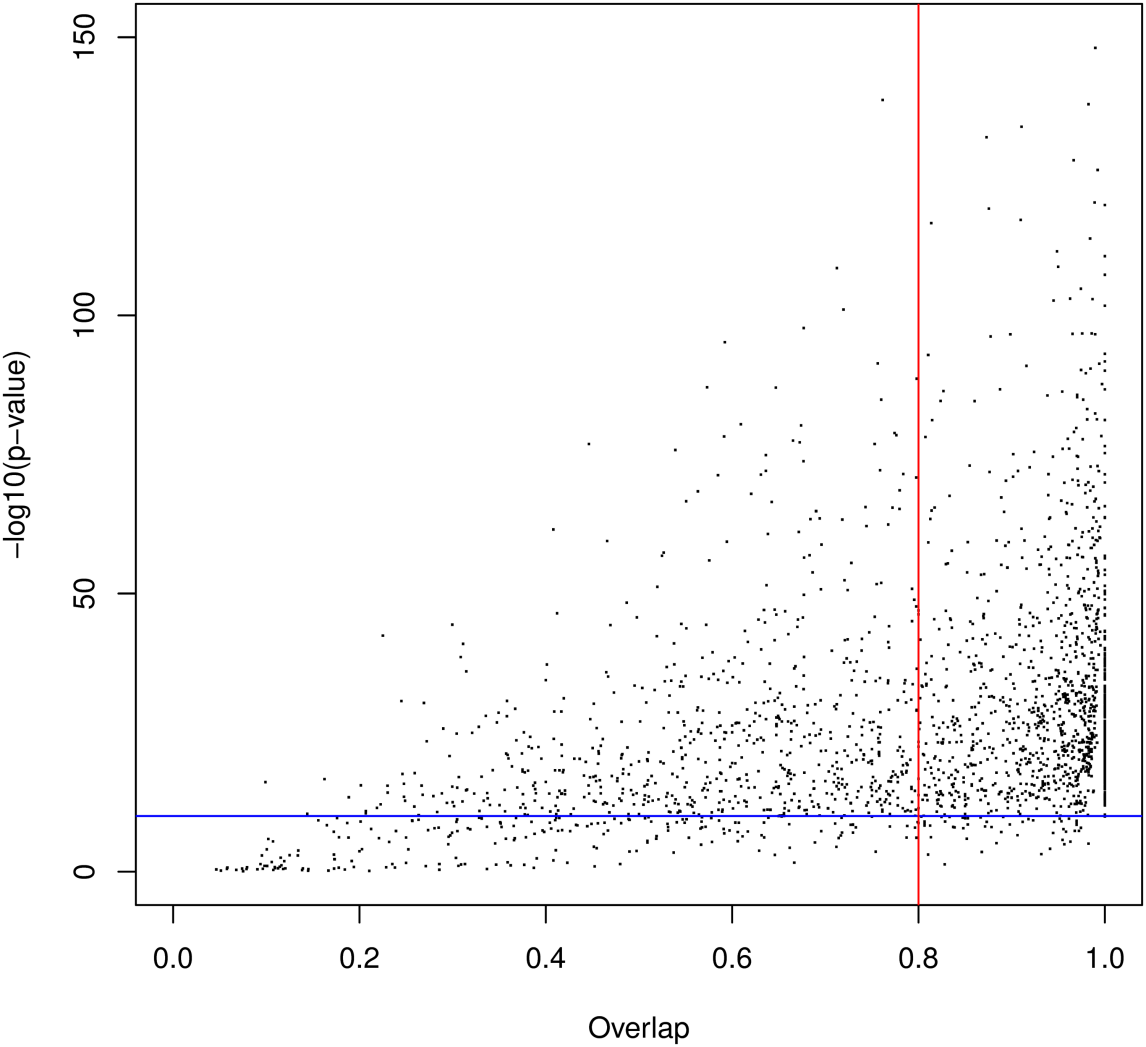
HMM/HMM comparison of domains identified by our approach that overlap a known Pfam domain. The x-axis shows the overlap ratio of the local alignment; the y-axis indicates the negative log of the alignment p-value; the blue line denotes the 10^−10^ p-value, while the red line denotes the 80% overlap.

We first ran our approach without integrating the known Pfam domains in the procedure (see Table 1). We used an e-value threshold of 10^−3^ for the BLAST search. With this cutoff, we identified a total of 10474 clusters (3905 with at least 5 hits). Among the 10474 clusters, 6489 have a p-value below 0.1% (3033 with at least 5 hits) on 2792 different proteins (1355 with a cluster with at least 5 hits). These clusters cover 32.85% of residues in the *P. falciparum* proteome. Clusters with at least 5 hits cover 12.90% of residues. The FDR of the procedure is estimated at 3.76% with the reverse sequences (see below for a comparison of the FDRs estimated with reverse sequences and 4-mer shuffling). Among the 3033 clusters with at least 5 hits, 2221 overlap an already known Pfam domain—only strong overlaps, *i.e.* greater than one third of the smallest domain, are considered here. We then assessed the extent to which our automatic procedure is able to recover the domains of the Pfam database. To answer this question, we generated the HMM associated with the 3033 clusters and we compared this HMM to that of the overlapping Pfam domain. We used HHsearch software [24] for this purpose (see Methods). Given two HMMs, this tool computes a local alignment of the HMMs with an associated p-value. From the HMM alignment, we also computed an overlap ratio by taking the ratio between the overlap length and the size of the longest HMM into account (see Fig 4). In most cases (87%), the HMM/HMM alignment had a significant p-value below 10^−10^, indicating that our HMM resembles (at least locally) the Pfam HMM. Moreover in 54% of cases, the HMM obtained with our cluster has an overlap with the Pfam HMM greater than 80%.

**Table 1:** Summary of the number of new domain occurrences identified by the approach. In the “Without Pfam integration” experiment, analyses were done on the entire genome, without masking the already known Pfam domain occurrences in the BLAST search nor using them as potential co-occuring hits in the co-occurrence analysis. On the contrary, in the “With Pfam integration” experiment the already known Pfam domain occurrences were masked in the BLAST search and used as potential co-occuring hits in the co-occurrence analysis.

Experiments	Without Pfam integration		With Pfam integration	
	all clusters	clusters ≥ 5 hits	all clusters	clusters ≥ 5 hits
number of clusters	10474	3905	7680	2279
with p-value ≤ 1%	6489	3033	6467	2240
proteins involved	2792	1355	3214	1293
residue coverage	32.85%	12.90%	24.61%	6.71%
estimated FDR	3.76%	3.85%	6.22%	1.83%
# domains overlapping a known Pfam-A occurrence	2830	2221	0	0

**Fig 5:**
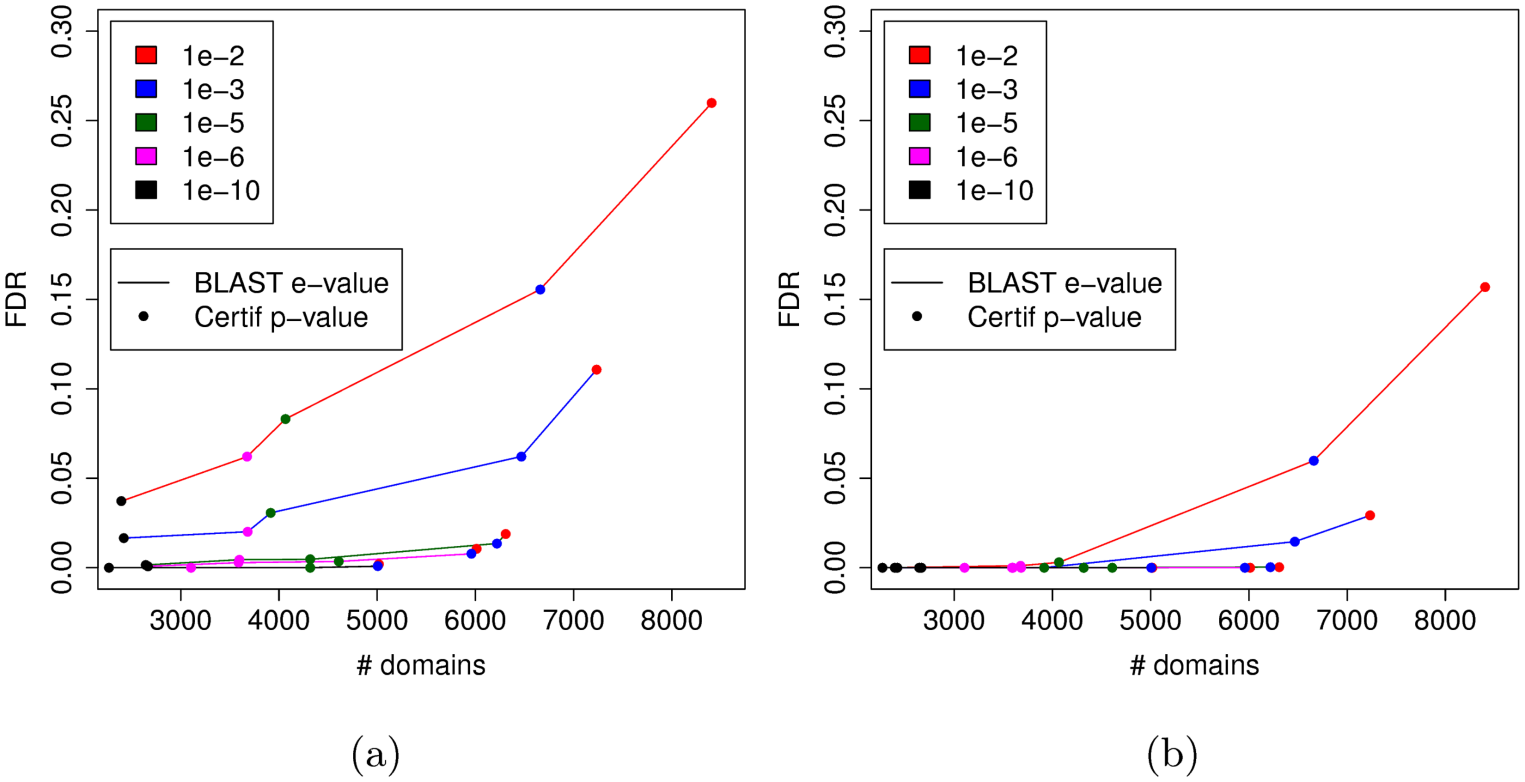
Number of domains and FDR obtained with different e-value and p-value cutoffs. This figure reports the FDRs estimated by the methods that use reverse sequences (a) and 4-mer shuffling (b).

We next challenged our approach for identifying new domains not already covered by a Pfam domain. As explained above, we included all known Pfam domains in the procedure, *i.e.* all *P. falciparum* protein regions covered by a Pfam domain were masked before the BLAST search and were used as potential co-occurring hits during the co-occurrence analysis. We ran our procedure with various thresholds for the BLAST e-value and for the p-value that assesses cluster relevance. Fig 5 reports the number of clusters and FDRs estimated with theses different thresholds, for the two FDR estimation procedures. As we can see on this figure the 4-mers procedure rapidly provides FDR estimates around 0%, which is likely very optimistic. For the rest of the paper, we thus consider only the FDR estimated with the reverse sequences, using a 10^−3^ cutoff for both the BLAST e-value and for assessing cluster relevance.

With these cutoffs, our method allowed us to identify a total of 7680 clusters (2279 with at least 5 hits; see Table 1). Among the 7680 clusters, 6467 have a p-value below 0.1% (2240 with at least 5 hits) on 3214 different proteins (1293 with a cluster with at least 5 hits). The MSAs of these domain families, and the HMMs that can be learned from the 2240 families with at least 5 hits are provided in the Supplementary Data. These clusters (which are not covered by Pfam domains) cover 24.61% of the residues in the *P. falciparum* proteome. Clusters with at least 5 hits cover 6.71% of residues. For comparison, Pfam domains cover 22% of this proteome. The FDR of the procedure is estimated at 6.22% (1.83% for the clusters with more than 5 hits). Clusters with a p-value < 0.1% gather a total of 121486 hits. Among these, 39617 have moderate e-values (> 10^−6^) and might not be considered in a classical whole-genome BLAST analysis. For comparison, the total number of BLAST hits with an e-value below 10^−6^ is 288351. Hence, with the 10^−6^ threshold, the co-occurrence property allowed us to increase the number of considered hits by 14%.

**Fig 6:**
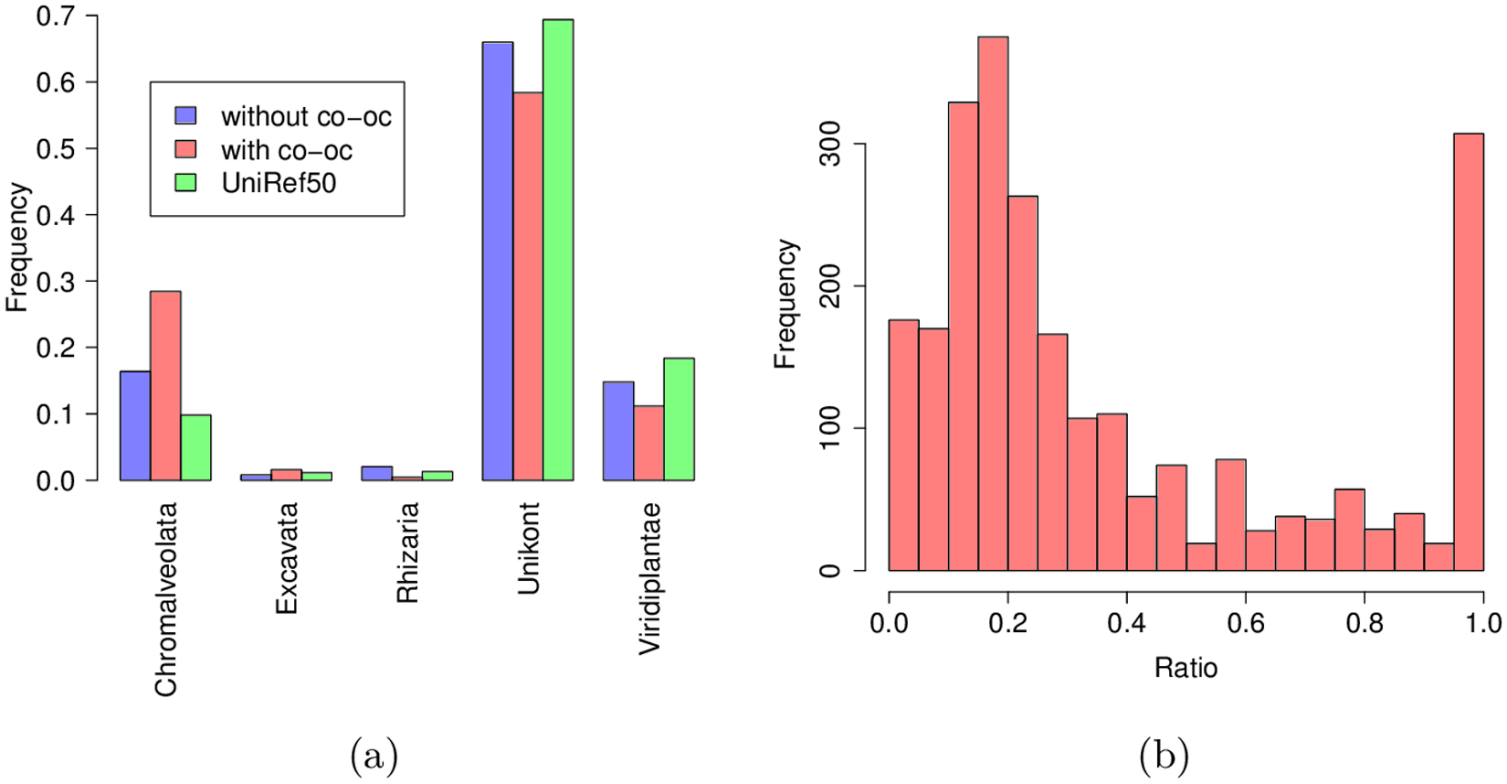
(a) Hit distribution among the five eukaryotic super-groups defined in Keeling et al. [25]. In green, the distribution in UniRef50 (restricted to eukaryotic sequences); in blue the distribution of all BLAST hits; in red the distribution of hits selected by co-occurrence. (b) Distribution of the proportion of Chromalveolate hits in the new families.

We first investigated the origin of the hits selected by our procedure, compared to all BLAST hits and the whole Uniref50 database. Each hit was classified according to its target species in the five super-groups covering Eukaryota: Chromalveolata, Excavata, Rhizaria, Unikont, and Viridiplantae [25]. Fig 6(a) reports the species distributions in all BLAST hits, only in the hits selected by our procedure, and in all proteins of the Uniref50 database. We observed slight enrichment of Chromalveolates in the BLAST hits compared to the Uniref50 database, and substantial enrichment of this super-group in the hits selected by co-occurrence. This was somewhat expected, considering that the proportion of false positives is likely lower for hits in the same super-group as *P. falciparum* than for hits outside this super-group. Moreover, this may also indicate that some of the newly discovered domains are specific to Chromalveolates. To assess this hypothesis, we computed the proportion of hits from Chromalveolates species for each of the 2240 domains (see Fig 6(b)). We found that 14% domains had a strong majority (> 90%) of hits from Chromalveolates species and hence could be considered as specific to this super-group.

#### Domain assessment

We next investigated the quality of the newly discovered domains. In all the following, we use the domains identified with the procedure integrating the already known Pfam domains. We used different quality measures (see below) and compared our results to the same measures applied to Pfam domain families. In order to compare families built on similar sequences, we restricted our analysis to Pfam families present on a *P. falciparum* protein, and the associated MSAs were restricted to sequences from Uniref50. Moreover, to avoid any bias due to the alignment step, Pfam families were realigned using the same tool and parameters we used to produce our models. Next, in order to assess the benefits of using co-occurrence information, we also conducted the same experiments on domains that were predicted using the total hit density instead of the co-occurring hit density. Namely, we used the same iterative procedure for identifying hit clusters, but applied it directly to the hit density (in blue on Fig 3).

**Fig 7:**
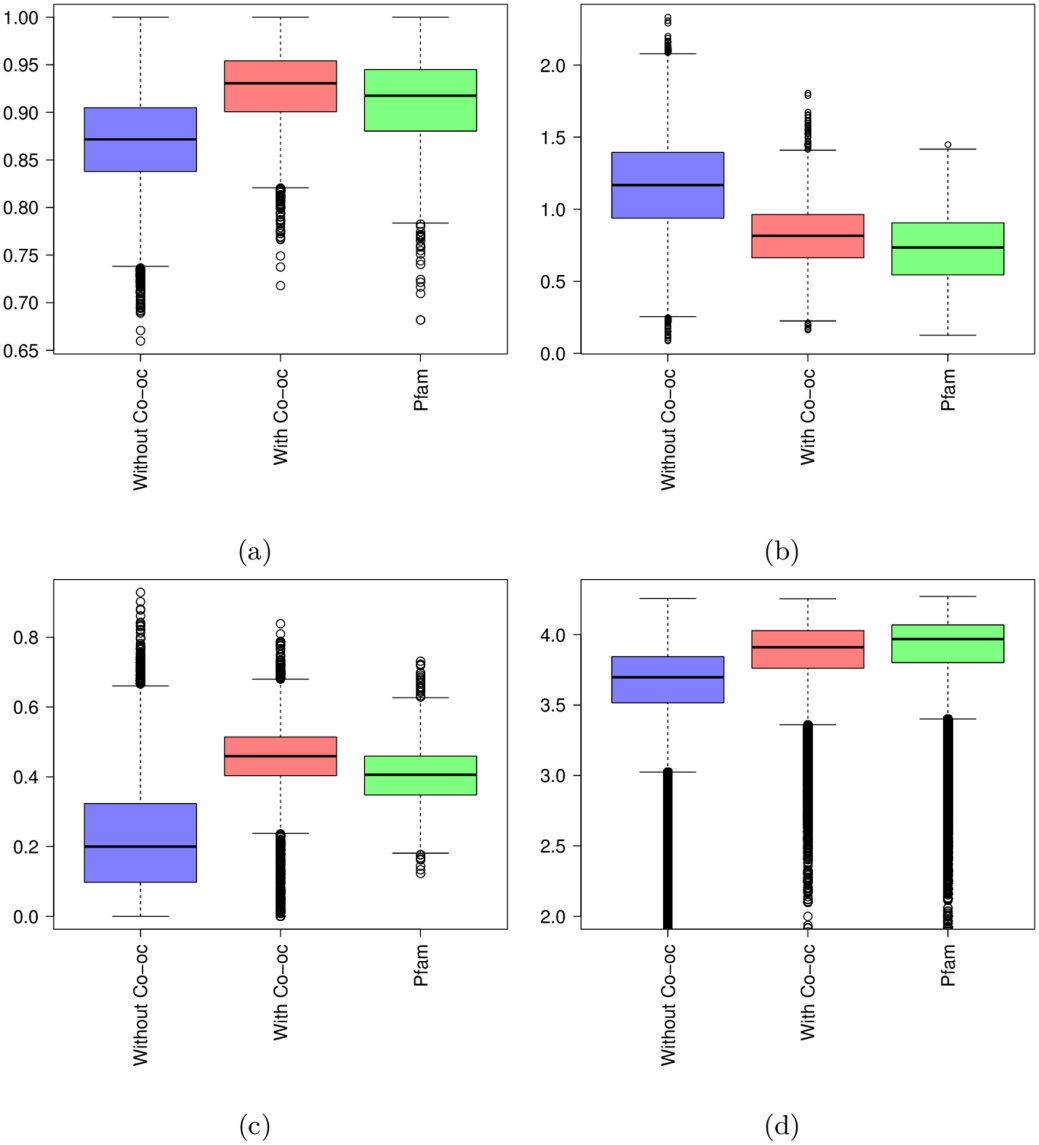
Quality scores measured on models obtained without co-occurrence (in blue), models obtained with co-occurrence (in red) and Pfam models (in green). (a) Homogeneity; (b) Entropy; (c) Hydrophobicity; (d) Complexity.

It is hard to assess the quality of a multiple sequence alignment in the absence of other information. We propose to use four types of measures for this (see Methods for details). The first measure (Fig 7(a)) is the alignment *homogeneity*. A good alignment usually has a minority of insertions and deletions on each position. A position has high homogeneity either if the residue proportion is very low (few sequences have an insert at this position) or high (few sequences have a deletion at this position). The second measure (Fig 7(b)) is the *entropy*, based on amino-acid classes, of match states in the alignment. In a good alignment, all residues on a match state tend to belong to the same amino-acid class and the entropy tends to 0. In a poor alignment, the distribution of residue classes is equi-probable and the entropy tends to ≈ 3. The third measure (Fig 7(c)) is the *hydrophobicity* score, which measures hydrophobic residue proportion at each position. Contrary to homogeneity and entropy it is difficult to establish what would be a “good” hydrophobic score. So here we will essentially compare our results to that of Pfam domains. The last measure (Fig 7(d)) is the *complexity* of sub-sequences in the MSA. Measuring the sequence complexity is a useful way of identifying repetitive sequences which are characteristic of non-globular regions [26].

As we can see on Fig 7, domain families identified with co-occurrence obtain quality scores relatively close to that of standard Pfam families. On the contrary, results obtained with all BLAST hits without the co-occurrence filtering are far from the standard Pfam families. This illustrates the interest of the approach and show that co-occurrence provides a useful way to filter out results of BLAST search at genome scale.

#### Comparisons with already known domain families

**Fig 8:**
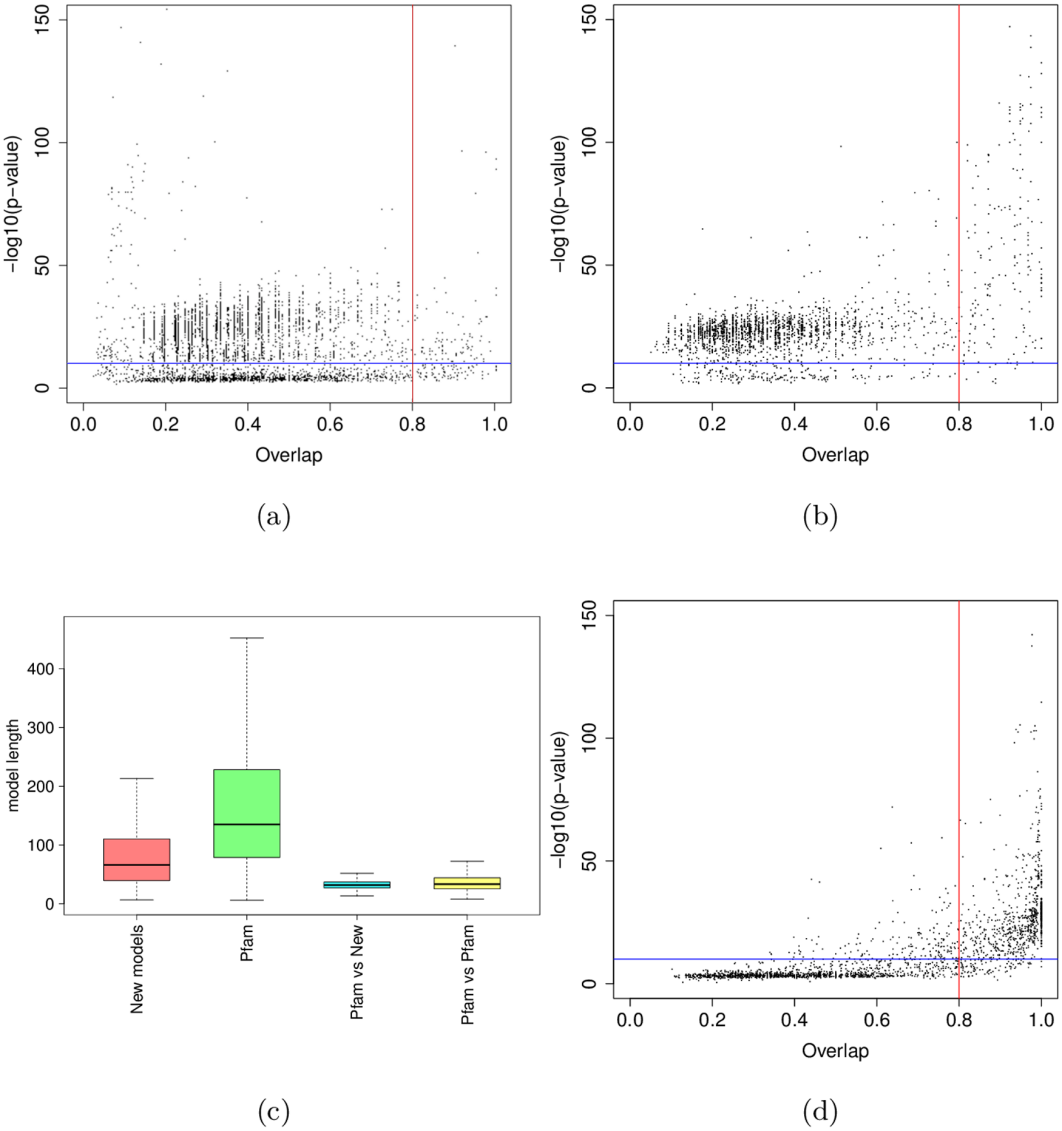
HMM/HMM comparison of new domain families and Pfam domain families. In Figures (a), (b) and (d), each point is associated with one particular HMM and corresponds to the best alignment found between this and all other HMMs. The x-axis shows the overlap ratio of the local alignment between the two HMMs; the y-axis indicates the negative log. of the alignment p-value; blue line corresponds to *y* = −*log*(10^−10^); while the red line corresponds to *x* = 0.8. (a) Pfam vs. Pfam comparison; (b) New families vs. Pfam comparison. Figure (c) shows the lengths of the models obtained by our approach (red), the lengths of all Pfam models (green), the lengths of the Pfam models associated with the points depicted in the top left quarter of figure (b) (blue), the length of the smallest Pfam model associated with the points depicted in the top left quarter of figure (a) (yellow).

We next assessed if some of the new domains were similar to known Pfam domain families that would not have been identified on the *P. falciparum* proteins (remember that the known Pfam occurrences have been masked in this experiment). We thus ran several profile/profile comparisons using HHsearch [24]. As above, we computed, for each HMM alignment, a p-value and an overlap ratio between the two HMMs. We first ran an all versus all comparison of Pfam HMMs. We identified, for each Pfam HMM, the other Pfam HMM that most resembles it. Fig 8(a) reports the p-values and overlap ratios of this analysis. As we can see, most Pfam HMM pairs have a p-value > 10^−10^ and/or an overlap ratio below 0.8. Hence we used these two cutoffs as a rough criterion to decide whether two HMMs are similar or not. Fig 8(b) reports the results of the comparisons between our new HMMs and Pfam HMMs. With the above criteria, we found that, on the 2240 models we produced, around 7% are strictly similar to a Pfam model and hence likely constitute an undetected occurrence of a known Pfam domain family. We noted however that a large part of our models get p-values below 10^−10^, indicating that they locally resemble a Pfam family. While such local resemblances is quite common between Pfam families themselves (see the numerous points in the top-left quarter of Fig 8(a)) it is possible that part of these new domains are actually partial occurrences of already known Pfam families. Genuine partial occurrences seems quite rare events [27], but it is known that alignment and annotation artifacts may result in partial domain observation [28]. To test this hypothesis, we compared the size of the new domain to that of the Pfam family that most resembles it. Surprisingly for the domains which have good p-value (< 10^−10^) but low overlap (< 80%) with a Pfam family, the new domain is longer than the Pfam family in 89% of the time. Moreover, most of the time the Pfam model is very short (see Fig 8(c)). Interestingly we observe the same type of short domains in the Pfam vs. Pfam comparison when restricting to domain pairs with good p-value and low overlap (Fig 8(c)). Hence, rather than partial domains, the newly identified domain could rather be viewed as extensions of smaller Pfam domains, something already quite common in the Pfam database.

To complement this analysis, we also compared our predictions to domain occurrences that can be identified with methods that use domain co-occurrence to complement Pfam HMM scores [5–9]. For this, we ran the dPUC2 [8] and DAMA [9] softwares on the *P. falciparum* proteins using default parameters. We removed all already known domain occurrences from the predictions and computed the coverage and FDR associated with the new domain occurrences identified by these approaches. DAMA and dPUC2 predict 1376 and 1039 new domain occurrences that do not overlap with already known domains (*i.e.* less than 30 residues or 50% overlap), respectively. The FDRs estimated by reverse sequences are around 4% and 2%, respectively. Of the 2240 new domains with more than 5 hits identified by our approach, 618 (27%) and 482 (21%) overlap by more than 30 residues (or 50%) a domain of DAMA or dPUC2, respectively. Note however that these overlaps are often partial and cannot be considered as strictly similar domains, which explains the difference with the 7% estimated above.

**Fig 9:**
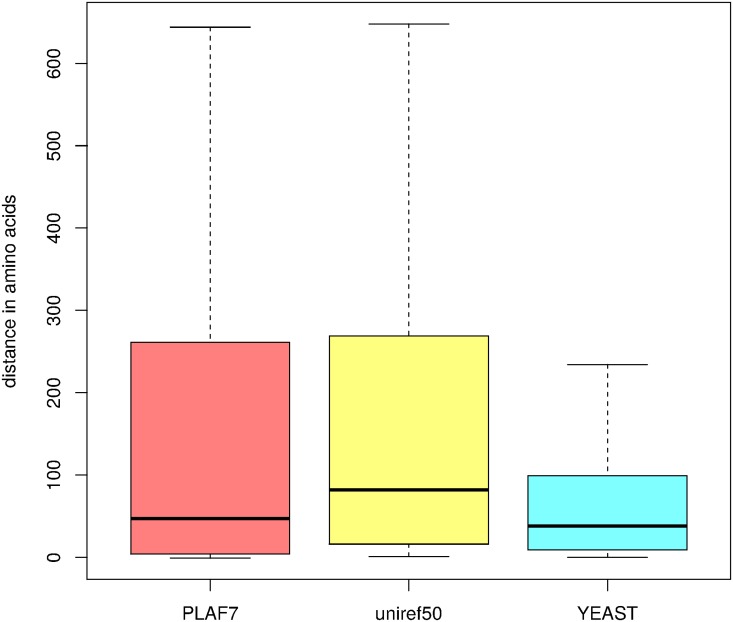
Distances (numbers of amino-acids) between adjacent domains. Red: distances between BLAST hits of newly identified domains and the closest annotated Pfam domain in *P. falciparum* proteins (only proteins with annotated Pfam domains were used in this analysis). Yellow: distances between BLAST hits and the same Pfam domain (if any) in the associated Uniref50 proteins. Blue: distances between adjacent Pfam domains in *S. cerevisiae*.

We next asked whether some of the new domains could not be real, entire, domains but elongations of already known Pfam domains. To assess this point, we computed for each domain the distance of the BLAST hits to the closest annotated Pfam domain on the *P. falciparum* protein sequence. Next, we computed the distance of the hits to the same Pfam domain (if any) on the Uniref50 proteins. For comparison, we also computed the distance between adjacent Pfam domains on a well annotated proteome (*Saccharomyces cerevisiae*). Results are summarized on Fig 9. As we can see on this Figure, hits that compose the domains are not particularly close to the already identified Pfam domains on the proteins (the median is around 50 amino-acids). On the contrary, those distances are larger than typical distances between adjacent domains in yeast.

#### Redundancy of new domain families

We next assessed the redundancy among our own predictions, *i.e.* we checked whether some of the new domains were similar to other newly discovered domains (the same family can be identified several times on a given proteome). To assess this hypothesis, we compared our models with each other using HHsearch (Fig 8(d)). 1454 (65%) of the new models did not seem to have close similarities with other new models, while 786 (35%) models were similar to one or more other new models. This higher proportion of redundancy compared to that observed against the Pfam database was somewhat expected, because domain families often have multiple occurrences in one proteome [29]. If we cluster together the models which have close similarities, we get an estimate of 1645 different families with more than 5 hits. Note that this is likely a conservative estimate, because our clustering algorithm grouped in the same class all models for which there exists a similarity relationship that is either direct or indirect (*i.e.* involving one or more other models).

#### Comparison against other automatically generated databases

We compared the results obtained by our procedure to Pfam-B and ProDom [30], two automatically generated domain databases. Pfam-B was part of the Pfam database until release 26. Actually, Pfam contained two types of domain families until this release: the high quality and manually curated Pfam-A families (“classical” Pfam domains, which are usually and until here in this article just called “Pfam”), and Pfam-B families, which were automatically generated by the ADDA algorithm [31] on the basis of all parts of Uniprot sequences not already covered by a Pfam-A occurrence. ProDom is a protein domain family database constructed automatically by clustering homologous segments with the MKDOM2 procedure based on recursive PSI-BLAST searches on the Uniprot database. Hence, both Pfam-B and ProDom families consists of aligned subsequences from the entire Uniprot database. Among the 460125 families contained in Pfam-B (release 26), we found 651 families with at least 5 different proteins of the UniRef50 database. These 651 families cover about 9.70% of the *Plasmodium falciparum* proteome. The ProDom database (December 2015 release) contains numerous very small families less than 30 amino-acids long. To be consistent with our experiments and the Pfam database, these families were not considered in the following. Among all 3739157 families contained in ProDom, we found 1453 families longer than 30 amino-acid, with at least 5 proteins of the UniRef50 database, one protein of *P. falciparum*, and that did not overlap with a Pfam-A domain. These families cover about 3.99% of the *P. falciparum* proteome. For comparison, clusters with at least 5 hits identified by our approach cover 6.71% of *P. falciparum* residues. Hence, in terms of coverage, our approach (6.71%) lies between ProDom (3.99%) and Pfam-B (9.70%).

**Fig 10:**
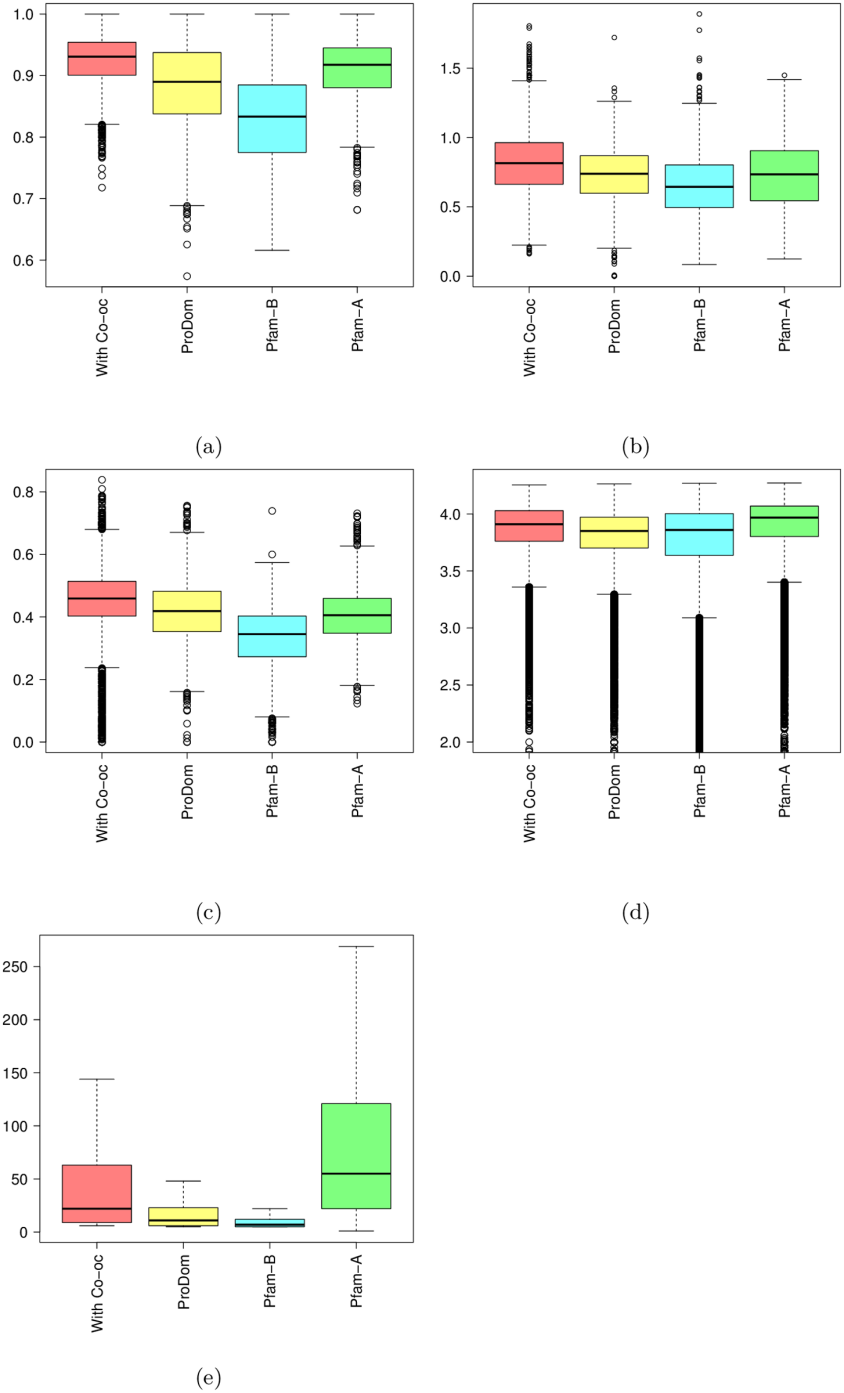
Quality scores (a-d) measured on families obtained by our approach (in red), ProDom families (in yellow), Pfam-B families (in cyan) and Pfam-A families (in green). (a) Homogeneity; (b) Entropy; (c) Hydrophobicity; (d) Complexity. Figure (e) shows the number of sequences in the families of the different databases.

The 651 Pfam-B and 1453 ProDom families were restricted to *P. falciparum* and UniRef50 proteins, and realigned using the same tool and parameters used to produce our models. Then, for each approach we computed the quality scores presented above (see Fig 10). As we can see, ProDom, and more importantly Pfam-B families, have very low homogeneity scores compared to Pfam-A families. This illustrates the fact that these databases sometimes include in one family different sequences that cannot be aligned and that should be clustered apart. On the contrary, they have good entropy scores, even better than that of Pfam-A families. This, however, is a mechanistic effect of the very low number of sequences that compose these families when they are restricted to UniRef50 sequences, as illustrated in Fig 10(e). For Pfam-B, for example, 80% of the considered families have less than 15 sequences in UniRef50. Hence, it seems that many families include only highly similar sequences, which obviously provides good entropy scores, but also induces a lack of diversity. On the contrary, by using co-occurrence information, our approach allows selection of more diverse sequences without the issue of introducing more false positives. In terms of hydrophobicity, ProDom has scores comparable to that of our approach and Pfam-A, while Pfam-B achieves slightly lower scores. Finally, ProDom and Pfam-B have comparable sequence complexity, but slightly lower than that of our approach and Pfam-A. Altogether, these results suggest that the domains built using co-occurrence are globally of better quality than those found in Pfam-B and ProDom. Note, however, that the purpose of these two databases is different from and somewhat wider than ours. Indeed, their goal is to build a library that pools all domain families of every species, while our aim is to find new domain occurrences for a specific species (here *P. falciparum*).

**Fig 11:**
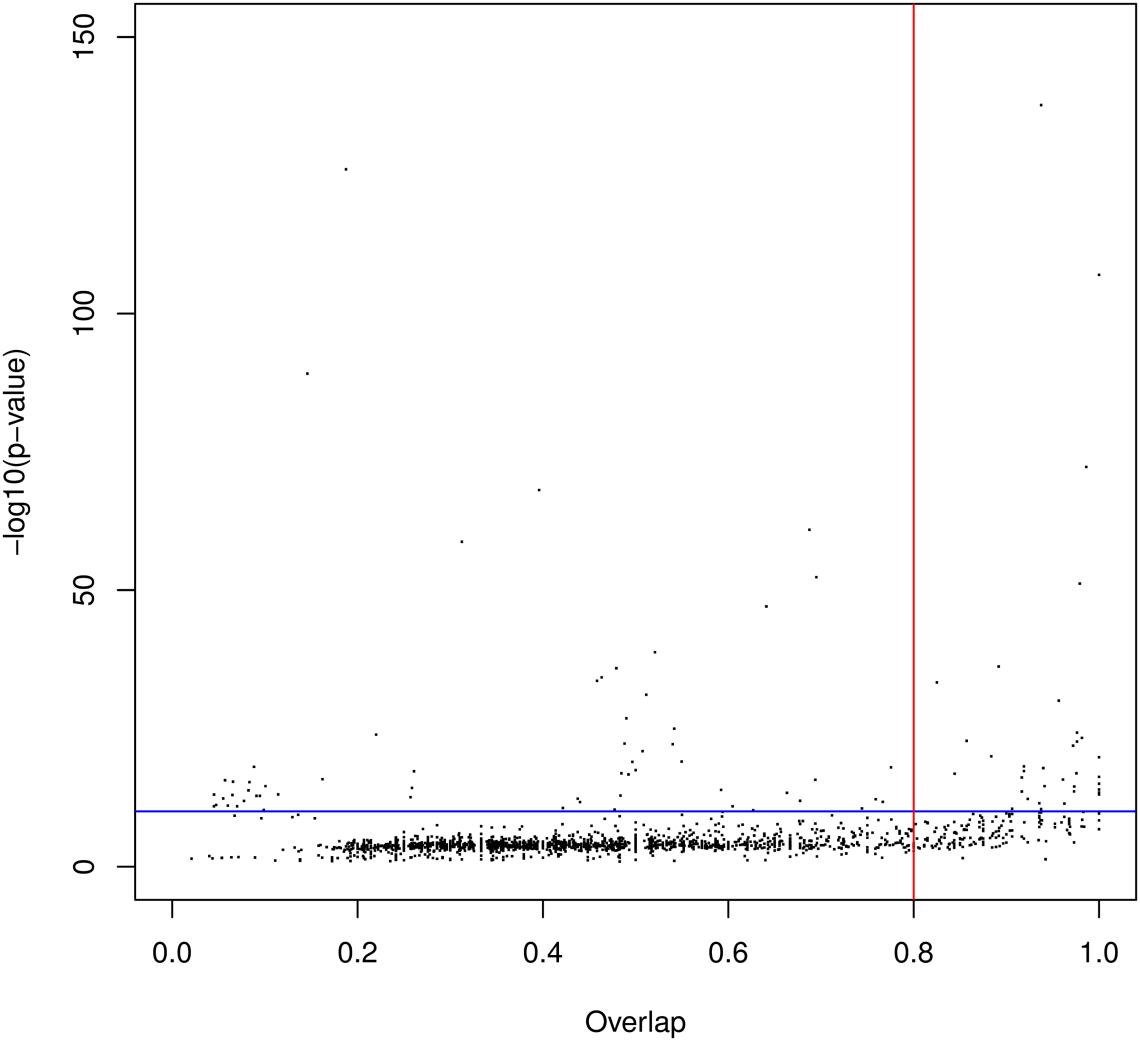
HMM/HMM comparison of new domain families *vs.* Pfam-B domain families. Each point is associated with one of our HMMs and corresponds to the best alignment found between this HMM and all Pfam-B HMMs. The x-axis shows the overlap ratio of the local alignment between the two HMMs; the y-axis indicates the negative log. of the alignment p-value; blue line corresponds to *y* = −*log*(10^−10^); while the red line corresponds to *x* = 0.8.

Finally, we directly compared our new domains to the Pfam-B families. As above, we used HHsearch to compare the HMMs learned from the families with more than 5 hits, to the HMMs provided by Pfam-B. Results are summarized on Fig 11. As we can see, most domains (95%) have low similarity with a Pfam-B family (p-value > 10^−10^ and overlap < 0.8).

#### Test against previous release

As an ultimate test, we ran our analysis using an older version of Pfam (release 26) and computed the number of new domains of the 28 release that this 26 analysis allowed us to recover. Over the 92 domain families with a hit on a *P. falciparum* protein and that are referenced in Pfam 28 and not in Pfam 26, we identified a domain on the same region for 54 of them. Careful inspection of these domains revealed that the new domains often have strong similarity with the domains identified by our approach. For example, the domain we identified on protein O77317_PLAF7 shows high similarity with the domain family PF16876 of the 28 release (p-value = 4 ⋅ 10*e*^−31^ and overlap = 0.99).

#### Gene ontology annotations

The Gene Ontology (GO) Consortium [32] provides a structured vocabulary describing gene functions according to three points of view (biological process, molecular function, and cellular component). Each ontology is organized as a directed acyclic graph where each node is associated with a term, while edges describe specialization and generalization relationships. The GO Consortium also provides a list of protein annotations for most sequenced organisms. With this information, we tried to associate an annotation with each new domain identified with at least 5 hits. For each domain family (cluster) we used the learned HMM to scan all UniRef50 proteins. All proteins with a p-value below 10^−10^ were added to the set of proteins from where the hits belong to (cluster members). Then, we gathered the annotations associated with all these proteins (without considering the query *P. falciparum* protein). We parsed the GO, and if more than 95% of annotated sequences shared the same GO term, we annotated the domain with this function. We set a minimum of 5 annotated sequences in a cluster to avoid irrelevant annotations. GO terms that are direct children of the root node were not considered as significant annotations and were ignored. Among the 2240 newly identified domains, we can thus propose an annotation for 1366 domains (see Supp. Data). Among these, for 1195 domains the proposed annotation is consistent with already known annotations of the *P. falciparum* protein where the domain has been identified. For 171 domains, the proposed annotations extend the known annotations.

**Fig 12:**
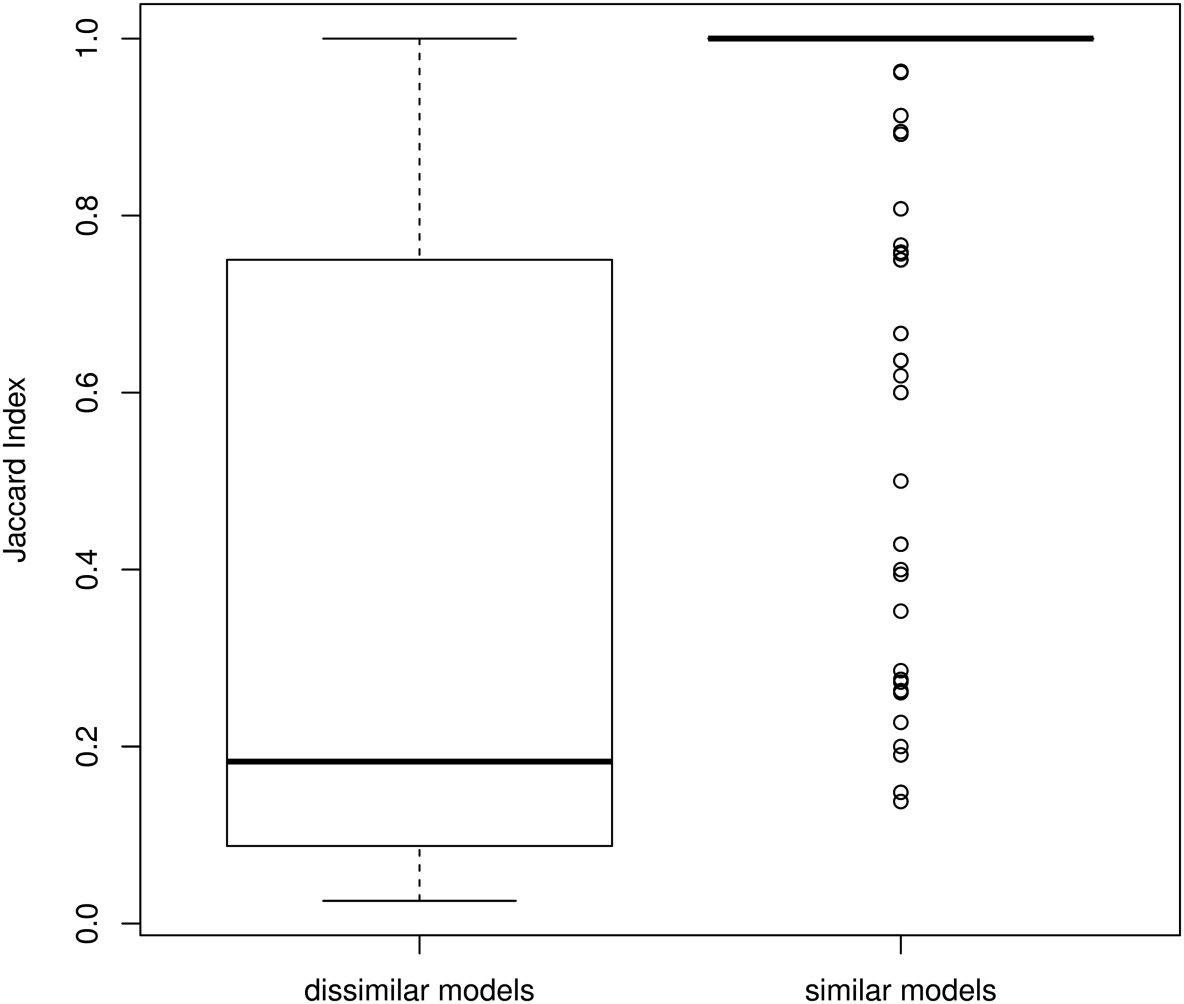
Similarity between GO annotations associated with domain families. Left: annotation similarity between dissimilar domain families. Right: annotation similarity between domain families identified as similar in Fig 8(d).

Finally, we checked whether the redundant new families (*i.e.* the 786 new families that resemble another new family) get similar GO annotations. To assess this point, we compared the annotations supplied to pairs of similar families, and the annotation supplied to pairs of dissimilar families. We used the Jaccard Index for measuring the similarity between two sets of GO annotations. Results are summarized on Fig 12. As we can see, similar families get GO annotations with similarity close to 1, while dissimilar families get much more different GO annotations.

## Discussion

Here we proposed a new method to take co-occurrence into account in a typical BLAST analysis and to construct new domain families on these results. Our method is based on the analysis of co-occurring hit density along the query protein. We designed a clustering procedure to identify clusters of similar hits that sign domain boundaries and a statistical test to assess the relevance of the identified clusters. Moreover, we have presented a procedure to estimate the proportion of false positives in a set of clusters.

We used *Plasmodium falciparum* as a case study to evaluate our approach. Our experiments showed an increase of 14% of the number of significant hits and an increase of 25% of the covered proteome. We identified 6467 significant hit clusters. The false detection rate was estimated at around 6% (3% for the clusters with at least 5 hits). We used these clusters to construct new domain families and to enrich databases of known domains. These models showed quality close to that of standard Pfam models and quite moderate redundancies with respect to original Pfam models, which indicates that they likely belong to new domain families that are not yet referenced in the database. Conversely, we identified more redundancies among the generated models themselves, which indicated the presence of multiple occurrences of a same domain family in the *P. falciparum* proteome.

Our approach could be improved in several ways. First, clustering is done by an ad-hoc procedure which involves two parameters that are set empirically. Hence an interesting improvement would be to integrate an automatic procedure able to find parameter values that best fit the protein at hand. Similarly, the quality of generated HMMs depends on the parameters of the alignment softwares (here BLAST and MUSCLE). We used standard parameter values in this study, but other parameters could certainly help in building better models. This is especially true for species like *P. falciparum*, which have amino-acid distributions far from the classical distribution observed in other species. Finally, the main drawback of our approach is that it cannot annotate all proteins of a given species. For *P. falciparum*, for example, our procedure is unable to identify any significant cluster in a total of 2151 proteins. The main reason for this is the existence of mono-domain proteins for which domain co-occurrence is of no use. In this case, it would be interesting to identify other features that could replace domains in the co-occurrence analysis. For example, tandem repeat sequences or disordered regions that constitute other classes of conserved protein sequences may be helpful in certain cases.

## Methods

### Identifying domains from BLAST hits

We used BLASTP software [15], release 2.2.28 with default parameters and a predefined e-value cutoff (for example 10^−3^). All hits smaller than 30 residues were removed, and in case of overlapping hits on a target protein only the hit with the lowest e-value was considered. Then hits were clustered using Algorithm 1. At the end of the of the process, each cluster corresponds to a putative domain.

**Algorithm 1**

**Input:** H: a set of co-occurrent hits on a query protein

**Output:** C: a set of hit clusters. Each cluster C∈C is defined by a set of hits and a start *C*_*s*_ and end *C*_*e*_ position on the protein.

 C←∅

 **do**

  $H^{*}\leftarrow H$ minus all hits overlapping any cluster C∈C

  Compute hits density with $H^{*}$

  *P* ← position with highest density

  *C* ← all hits covering *P*

  *C*_*s*_ ← lowest starting position in *C*

  *C*_*e*_ ← highest ending position in *C*

  **do**

   Unstable ← False

   *N* ← *C* size

   Compute *Ns* and *Ne*, the number of hits in *C* covering *C*_*s*_ and *C*_*e*_, respectively

   **if** *Ns* < 1/3 ⋅ *N* **then**

    *C*_*s*_ ← *C*_*s*_ + 1

    Unstable ← True

   **end if**

   **if** *Ne* < 1/3 ⋅ *N* **then**

    *C*_*e*_ ← *C*_*e*_ − 1

    Unstable ← True

   **end if**

   Remove hits in *C* if more than 30% of residues outside range [*C*_*s*_ … *C*_*e*_]

  **while** Unstable

  **if** (*C*_*e*_ − *C*_*s*_) > 30 **then**

   Add *C* in C

  **end if**

 **while** at least one new cluster in C

### Computational complexity and computing time

If *n* is the total number of hits returned by BLAST for a query protein, identifying the co-occurrent hits and computing the co-occurrent hit density (red curve) takes *O*(*n* log *n*) operations. Then, if *n*′ is the number of co-occurrent hits on the protein, the above algorithm takes *O*(*n*′ ⋅ *q*) operations, with *q* the total number of clusters on the protein. *q* is expected to be low (a dozen or less) and *n*′ << *n*, so the total time complexity is *O*(*n* log *n*). On the *P. falciparum* experiments and standard laptop (core i7, 8GO RAM), with BLAST e-value cutoff of 10^−3^ the identification of co-occurrent hits on all proteins takes 7 min, while cluster identification takes 29 sec. P-value computation takes 33 sec. When we include the time needed for read/write operations, the total computing time for analyzing this proteome (once the BLAST analysis has been done) is around 17 min.

### HMM learning

HMM are trained using HMMER3 software (release 3.1b2) with the following command:

hmmbuild -n <hmm_name> –amino –fast <hmmfile_out> <msafile>;

with <hmm_name> a name for the trained HMM, –amino specifies that input alignement is protein sequence data, –fast assigns columns with >= 50% (default) residues as match position, <hmmfile_out> the output HMM file, <msafile> the input alignement.

### HMM/HMM comparisons

HMM-HMM comparisons are done using the HHsearch software provided in the HHsuite (release 2.0.16) with the following command:

hhsearch -i <hmmfile_in> -d <hmm_db> -o <resfile_out> -loc

with <hmmfile_in> the input HMM, <hmm_db> the HMM database to compare to, <resfile_out> the file containing results, -loc to search the best local alignement.

### Assessing domain quality

Assessing the quality of a multiple sequence alignment is not easy in the absence of other information. We propose to use four types of measures for this. The first measure is the alignment *homogeneity*. A good alignment usually has a minority of insertions and deletions on each position. This can be measured by the proportion of residues on each position. Namely, this proportion should be either very low (when a few sequences have an insert at this position) or high (when a few sequences have a deletion at this position). We measure the homogeneity by

$$\begin{array}{c} \text{Homogeneity}=\frac{1}{p\times s}\sum_{i=1}^{p}Max\left(r\left(i\right);\bar{r}\left(i\right)\right), \end{array}$$ (3)

with *s* and *p* representing the number of sequence and the length of the MSA, respectively, while *r*(*i*) and $\bar{r}\left(i\right)$ represent the number of residues and *indels* (insertion or deletion) at position *i*, respectively. With this formula, a good alignment should have a score that tends to 1.

The second measure is the *entropy* of match states in the alignment. Entropy is based on amino-acid classes. Briefly, amino acids can be grouped according to physicochemical features. We used the same definition of classes as in the Seaview software package [33].

$$\begin{array}{c} \text{Entropy}=\frac{1}{m}\sum_{i\in \text{MatchStates}}\sum_{c\in \text{Classes}}-p_{c}\left(i\right)\log_{2}\left(p_{c}\left(i\right)\right), \end{array}$$ (4)

with *m* representing the number of match states in the MSA. We consider a position as a match state if the number of residues at the position is higher than the number of indels. *p*_*c*_(*i*) represents the proportion of residues belonging to class *c* at position *i*. In a good alignment, all residues on a match state tend to belong to the same amino-acid class and the entropy tends to 0. In a poor alignment, the distribution of residue classes is equi-probable and the entropy tends to ≈ 3.

The third measure is the *hydrophobicity* score:

$$\begin{array}{c} \text{Hydrophobicity}=\frac{\text{Number}\;\text{of}\;\text{hydrophobic}\;\text{match}\;\text{states}}{\text{Number}\;\text{of}\;\text{match}\;\text{states}} \end{array}$$ (5)

We consider a match state as hydrophobic if the majority of residues at this position are considered as hydrophobic (residues L, A, F, W, V, M, I, P, C and G). The hydrophobic residue proportion is commonly used as a measure of globularity, because globular domains have a stable amount of strong hydrophobic amino acids (about one third of the sequence) [34]. Note that contrary to homogeneity and entropy it is difficult to establish what would be a “good” hydrophobic score. So here we will essentially compare our results to that of Pfam domains.

The last measure is the *complexity* of sub-sequences in the MSA.

$$\begin{array}{c} \text{Complexity}=\sum_{r\in \text{amino}\;\text{acids}}-p_{r}\log_{2}\left(p_{r}\right), \end{array}$$ (6)

with *p*_*r*_ representing the relative frequency of amino acid *r* in the sequence at hand. Contrary to the three previous measures, which are based on the columns (positions) of the alignments, this one is applied on each sequence independently. Measuring the sequence complexity is a useful way of identifying repetitive sequences which are characteristic of non-globular regions [26].
